# PYHIN proteins: guardians or contributors to hepatitis B virus pathogenesis?

**DOI:** 10.3389/fimmu.2026.1953996

**Published:** 2026-09-18

**Authors:** Wen-Qiang He, Min Li, Jun-Feng Li, Li-Ting Zhang

**Affiliations:** 1 Department of Hepatology and Infectious Disease Research Laboratory, Lanzhou University First Hospital, Lanzhou, China; 2 First Clinical Medical College of Lanzhou University, Lanzhou, China; 3 Institute (Center) of Portal Hypertension Research, Lanzhou University First Hospital, Lanzhou, China

**Keywords:** AIM2, hepatitis B virus, IFI16, interferon-stimulated genes, PYHIN

## Abstract

Human pyrin and HIN domain (PYHIN) family proteins play central roles in the host defense against viral infection. These PYHIN proteins, including IFI16, AIM2, MNDA and PYHIN1, mainly function as intracellular DNA sensors. Among them, IFI16 is supported by the most robust mechanistic evidence, including direct binding to hepatitis B virus (HBV) covalently closed circular DNA (cccDNA) in the nucleus and subsequent suppression of viral transcription. They recognize HBV DNA in different cellular substructures, triggering innate immune responses and interfering with steps of the viral life cycle. This article reviews the latest research progress of the PYHIN protein in HBV infection, and summarizes its immunoregulatory function and its role in the disease progression. We focus on the important question of the dual antiviral and pro-damage functions of PYHIN. On one hand, PYHIN has the ability to sense HBV DNA and inhibit the replication of the HBV. On the other hand, the host response mediated by PYHIN may also promote chronic inflammation, genomic instability and tumor formation. Furthermore, we collated the mechanism by which HBV evaded inflammasome activation by suppressing PYHIN expression through HBx. Here, we have emphasized the necessity of conducting in-depth mechanism research to clarify the role of the PYHIN family proteins in HBV infection contexts, and proposed the feasibility of implementing precise targeted immunotherapy based on the functions of PYHIN, aiming to provide strategies for achieving hepatitis B cure.

## 1 Introduction

According to data from the World Health Organization (WHO), approximately 250 million people worldwide are infected with the hepatitis B virus (HBV), and more than 800,000 people die each year due to liver diseases related to hepatitis B (1, 2). HBV infection is a cause of progression to liver fibrosis, cirrhosis, and hepatocellular carcinoma (HCC) (3). While current antiviral therapies can effectively inhibit HBV replication, they are unable to eliminate the covalently closed circular DNA (cccDNA) and integrated DNA. Once therapy is stopped, the viral replication resumes (4–6). Similarly, pegylated interferon (IFN) has shown excellent antiviral effects for some patients; its applicability is limited, and the induction of numerous of IFN-stimulated genes (ISGs) is associated with serious adverse reactions (7, 8). Therefore, in order to achieve the clinical cure of hepatitis B in most patients, it is necessary to find new treatment strategies.

Studies have shown that host antiviral factors have a high potential for antiviral activity, capable of inhibiting the replication of HBV at multiple stages of its life cycle, and intracellular DNA sensors play a crucial role in this process (8, 9). The pyrin and HIN domain (PYHIN) family is a host gene that recognizes HBV DNA and cccDNA, yet the exact function and the virus evasion mechanism of this family remain unclear (10–12).

It is worth noting that under steady-state conditions, the expression of the endogenous IFN gene activation factor (STING) in liver cells is relatively low (13), and the DNA sensing ability is limited. Although this defect is beneficial for HBV infection, it does not deny the importance of the PYHIN family proteins in the reactivation process. Type I IFN from non-endothelial cell sources can upregulate the pattern recognition receptor (PRR) pathway in liver cells, thereby achieving inducible DNA sensing (14). Moreover, hepatocytes constitutively expressing PYHIN proteins can detect HBV nucleic acids and trigger inflammatory responses (12). Additionally, PYHIN proteins can exert antiviral effects independently of the STING pathway (11, 15).

This review aims to summarize the research progress of the human PYHIN gene family in the host’s resistance to HBV and disease progression, explore the functional differences among PYHIN family members and their potential roles in liver tissue damage, and analyze the feasibility of developing new therapeutic strategies based on the PYHIN pathway. Here, we have distinguished between the mechanisms that have been directly confirmed in HBV infections, the results inferred from other DNA viruses or non-hepatitis systems, and the hypotheses that require experimental verification.

## 2 Overview of the structure and function of the PYHIN proteins family

### 2.1 Structure and expression pattern of PYHIN proteins

The PYHIN proteins family members share a conserved domain architecture characterized by an N-terminal PYD (pyrin) domain and one or two C-terminal HIN (hematopoietic IFN-inducible nuclear antigen) domains, which enable recognition of double-stranded DNA (dsDNA) and activation of downstream immune signaling (11, 16). Human PYHIN members include IFN-induced protein 16 (IFI16), absent in melanoma 2 (AIM2), myeloid cell nuclear differentiation antigen (MNDA), and PYHIN1 (also known as IFIX) (11) (**Figure 1A**). The amino acid sequence similarity of the PYD domain in the human PYHIN family is only about 17%, while the similarity of amino acid sequences in different HIN domains is approximately 50% (**Figures 1A, B**). IFI16 functions as a nuclear and cytoplasmic DNA sensor that activates IFN signaling pathways (14, 15); AIM2 acts as a cytoplasmic inflammasome receptor detecting dsDNA, promoting IL-1β and IL-18 secretion (16); MNDA is mainly found in myeloid cells. The MNDA HIN domain has been shown to interact with dsDNA through a structurally unique binding mode (17); and PYHIN1 participates in antiviral defense as an IFN-inducible DNA-binding protein (11, 18, 19).

**Figure 1:**
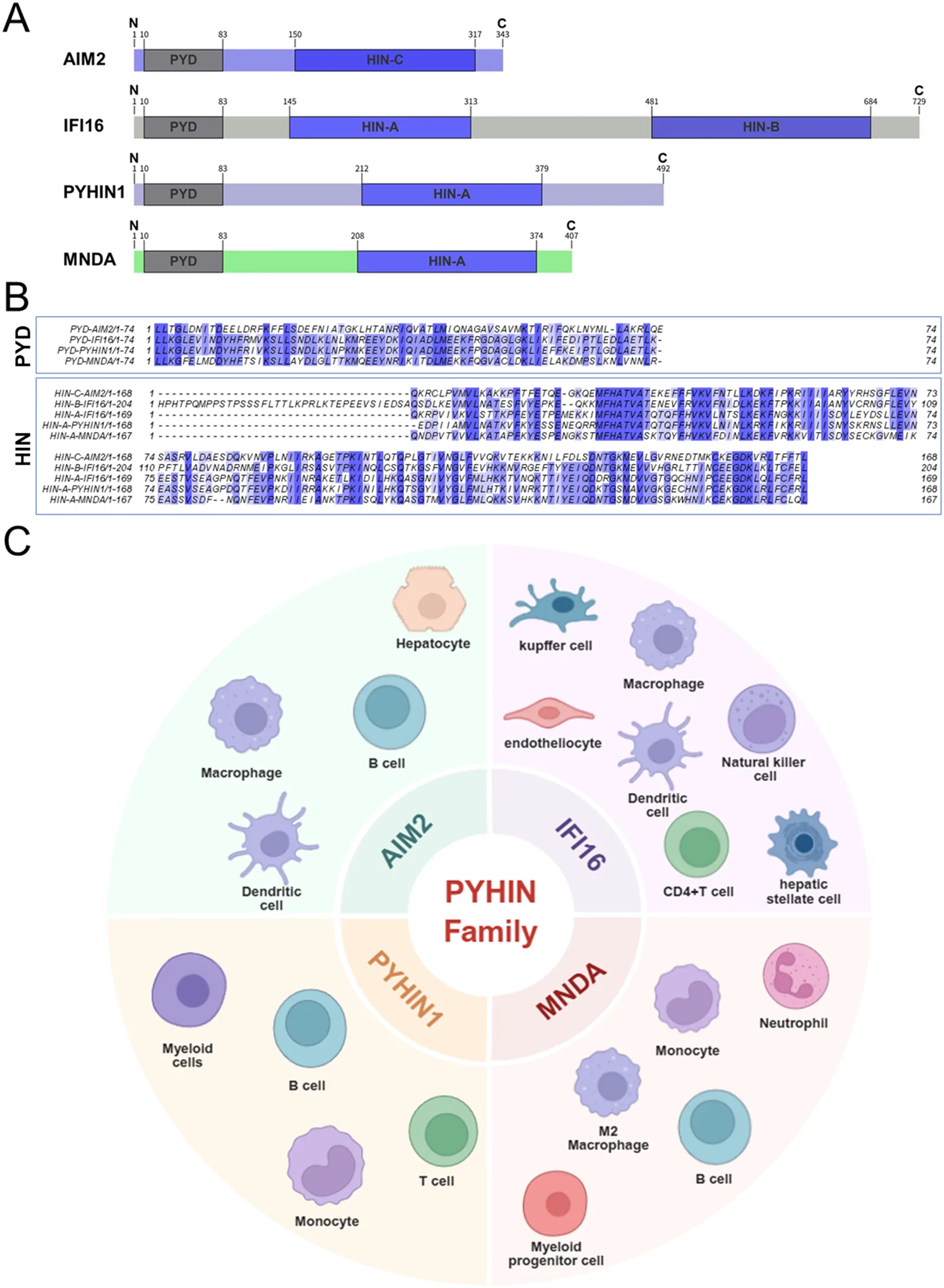
Structural characteristics of PYHIN proteins. **(A)** Domain architecture diagram of human PYHIN family. **(B)** The amino acid sequences of the PYD and HIN domains of the PYHIN family. **(C)** Major expression cell of the PYHIN proteins.

In the liver, the baseline expression of the PYHIN family members is usually low in liver cells, but it is induced in response to inflammatory stimuli or viral infections (13, 16). It is worth noting that IFI16 is expressed in the nuclei of kupffer cells, endothelial cells, natural killer cells, dendritic cells and hepatic stellate cells in both healthy donors and patients with chronic HBV infection (CHB) (20). AIM2 is expressed in hepatocytes and its expression is upregulated upon HBV infection, positively correlating with the severity of liver inflammation (21). Expression profiles of PYHIN proteins exhibit cell-type specificity.

In immune cells, they are broadly expressed in myeloid and lymphoid lineages: MNDA is predominantly expressed in myeloid cells (monocytes, macrophages, neutrophils) and also detectable in B cells (15, 22, 23); MNDA is expressed in hepatic myeloid cells, particularly in tumor-associated M2 macrophages, where it promotes M2 polarization and facilitates HCC metastasis (24). MNDA was found to be capable of regulating the transcriptional activity of monocytes (25). IFI16 is present in macrophages and CD4+ T cells (26, 27); and The AIM2 gene is highly expressed in innate immune cells, particularly in macrophages, B cells and dendritic cells (28) (**Figure 1C**).

This differential expression between liver cells and immune cells reflects the diverse tissue-specific functions of this family in DNA sensing and immune surveillance.

### 2.2 The classical signaling pathways and main functions of the PYHIN family genes

Members of the PYHIN family trigger different signal cascades by recognizing pathogen DNA, mainly involving two classical pathways: The first pathway is the IFI16-STING pathway (29, 30). IFI16 is predominantly a nuclear protein that detects viral dsDNA within the nucleus via its HIN domain (20, 31). Upon recognition of nuclear viral DNA, IFI16 initiates a nucleus-derived signal that is propagated to the cytoplasmic STING signaling hub (32); STING then undergoes oligomerization on ER-derived membranes, recruiting and activating TANK-binding kinase 1 (TBK1) through trans-autophosphorylation. Activated TBK1 phosphorylates interferon regulatory factor 3 (IRF3), which dimerizes and translocates to the nucleus, inducing the expression of type I interferons (IFNs) and ISGs, thereby establishing an antiviral state (33, 34). After AIM2 recognizes intracellular dsDNA, it interacts with the adaptor protein ASC through its PYD domain, recruits and activates caspase-1, thereby promoting the maturation and secretion of pro-inflammatory cytokines such as interleukin (IL)-1β and IL-18, and inducing pyroptosis to limit the replication and spread of pathogens (34, 35). In addition, members of the PYHIN family also possess various non-classical functions. IFI16 can be involved in transcriptional regulation, epigenetic regulation and cell cycle regulation, suggesting that it plays multiple roles in maintaining the homeostasis of host cells and immune regulation (**Figure 2**).

**Figure 2:**
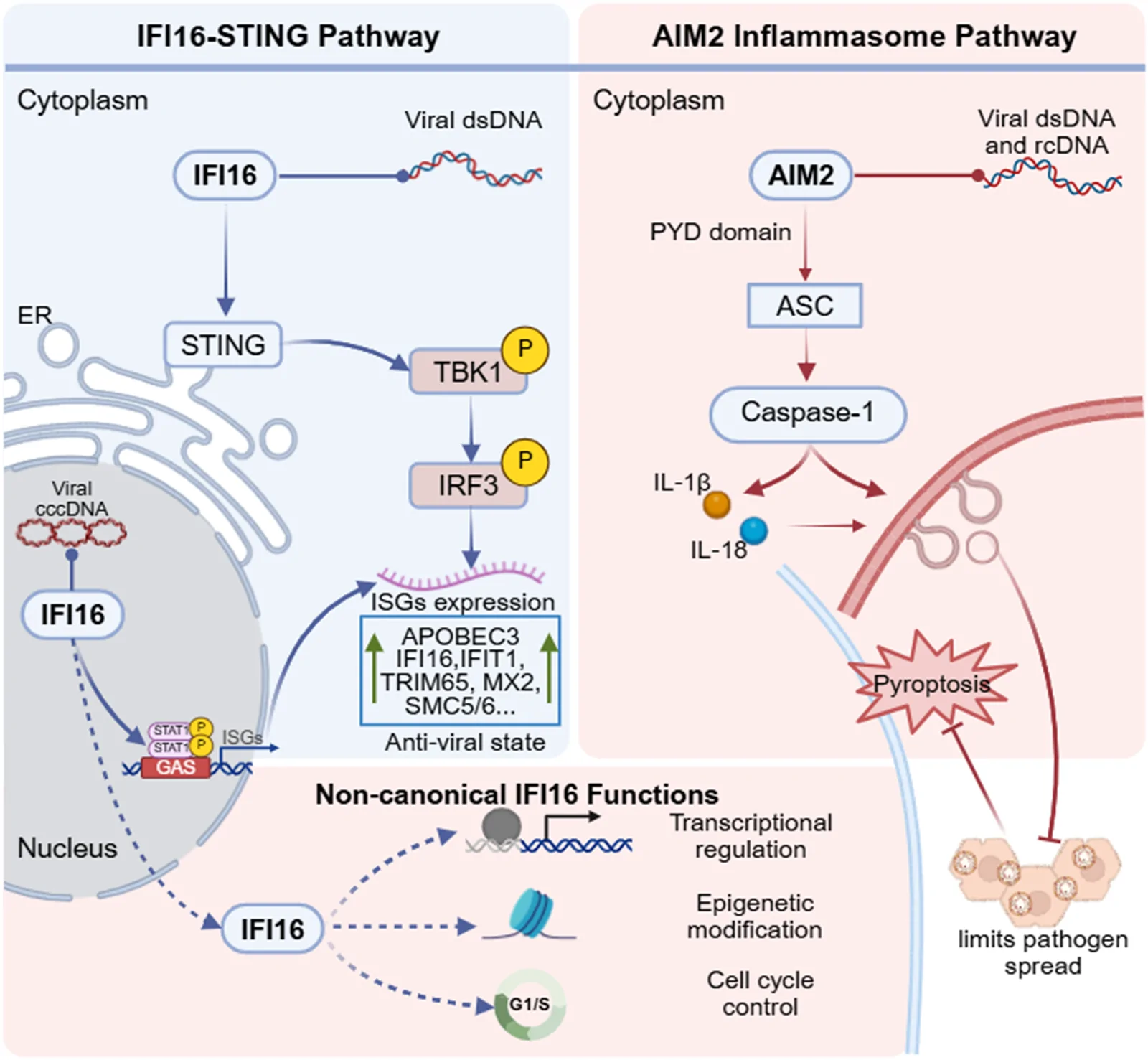
Schematic overview of IFI16- and AIM2-mediated DNA sensing and innate immune signaling (1). In the cytoplasm, IFI16 recognizes viral dsDNA and activates the STING-TBK1-IRF3 pathway, thereby upregulating the expression of ISGs (such as APOBEC3, IFI16, IFIT1, TRIM65, MX2), to establish an antiviral state. In the cell nucleus, IFI16 can sense viral double-stranded cDNA and induce the expression of ISGs (2). In the cytoplasm, AIM2 can detect viral double-stranded DNA and the reverse strand DNA, and recruit ASC through its PYD domain, thereby activating Caspase-1. This leads to the maturation of IL-1β/IL-18 and induces programmed necrosis to limit the spread of pathogens (3). IFI16 in the cell nucleus also participates in transcriptional regulation, epigenetic modification, and the regulation of G1/S cell cycle transition. Solid lines represent experimentally verified interactions. Dashed lines represent the mechanisms inferred based on other virus or indirect evidence. This image was created using BioRender.

The PYHIN family is one of the important immune barriers for the host against DNA viruses and retroviruses (12). Numerous studies have shown that IFI16 and AIM2, after recognizing viral DNA, can restrict the replication and spread of the virus through the aforementioned signaling pathways (16). Specifically, IFI16 plays a crucial role in limiting the replication of herpes simplex virus, human papillomavirus, and human immunodeficiency virus during infection (36–38); AIM2 mainly recognizes viral DNA in the cytoplasm and performs particularly well in resisting pathogens such as murine cytomegalovirus (39). Moreover, members of the PYHIN family can mediate antiviral effects through various means such as directly binding to the viral genome, interfering with viral gene expression, and activating the innate immune system (36, 40). While the members of the PYHIN family differ in their virus recognition mechanisms and signal transduction pathways, they collectively form an important part of the host’s early antiviral immune response.

Beyond these classical pathways, IFI16 also regulates transcription, chromatin remodeling, and cell cycle progression, highlighting its multifunctional roles in host immunity (29, 34, 36).

## 3 PYHIN-mediated recognition of HBV and antiviral effector functions

### 3.1 Identification of HBV DNA and cccDNA by IFI16 and AIM2

In HBV infection, PYHIN family proteins act as important PRR that recognize HBV DNA during early infection (12, 41). Among them, IFI16 is localized in the nucleus, where it directly binds to HBV cccDNA via its HIN domain, and also recognizes dsDNA intermediates in the cytoplasm, thereby functioning as a dual-phase intracellular DNA sensor (42). Specifically, IFI16 accumulates in the viral chromatin region by binding to cccDNA, which stably exists as a viral minichromosome in the nucleus, and participates in regulating the formation of the viral transcription complex (40). In parallel, its recognition of HBV DNA intermediates prior to nuclear entry allows IFI16 to trigger innate immune responses at multiple stages of infection (43).

In contrast, AIM2 is predominantly cytoplasmic and recognizes dsDNA structures, this function is well established in HBV and Poxvirus systems (41, 44, 45). Following viral entry, HBV rcDNA undergoes repair to form cccDNA, a process that may expose incomplete dsDNA structures, potentially enabling AIM2 recognition. However, direct mechanism evidence supporting AIM2-mediated recognition of HBV DNA remains limited, with most findings based on speculation or indirect observations.

### 3.2 Inducing of type I interferon and inflammasome responses

PYHIN protein plays a role in regulating innate immune signals during HBV infection, but the strength of evidence varies among different members (43, 46). In HBV-infected cells, nuclear IFI16 can activate type I IFN production and ISGs via STING and downstream pathways, producing indirect anti-HBV effects because ISGs impair multiple steps of the HBV life cycle, such as the RNA stability, nucleocapsid assembly and virus release (40, 46). Clinical correlations show IFI16 expression associates with HBV-related inflammation, indicating contributions to both antiviral defense and pro-damage functions (47); however, whether this association reflects a causal role remains to be established.

Evidence from an HBV/HCV coinfection model in human monocytes suggests AIM2 inflammasome activation in response to HBV-associated infection, whereas direct AIM2-mediated recognition of HBV DNA in HBV infection remains insufficiently established (48). The expression level of AIM2 is higher in patients with acute hepatitis B, and the expression level of AIM2 is positively correlated with the content of IL-1β and IL-18 in serum (49). In CHB infection, the elevation of AIM2 is associated with increased inflammation, as shown in clinical studies (50). In experimental models of HBV and HCV coinfection, HBV has been demonstrated to activate the AIM2 inflammasome in monocytes, leading to IL-18 production, which in turn induces IFN-γ expression by NK cells (41). In addition, the activation of AIM2 inflammasome may promote clearance of infected cells, whereas excessive activation could lead to liver inflammation and damage (40, 44, 48), suggesting that its function may depend on the intensity and duration of activation; however, direct experimental evidence in HBV infection remains lacking. MNDA, as an ISG, can be induced by type I IFN and is essential for monocyte IFNα induction (51). Unlike AIM2 and IFI16, MNDA does not act as a cytosolic dsDNA sensor triggering innate immune signaling; although its HIN domain retains the structural capacity to bind dsDNA (17, 24), its immunological function centers on transcriptional regulation rather than pathogen DNA sensing. Specifically, MNDA is required for the induction of IRF7 by all type I IFN inducers tested (22, 52). Mechanistically, MNDA is recruited to the IRF7 promoter following IFNAR stimulation and is essential for the recruitment of STAT2 and RNA polymerase II to this locus (52). MNDA is thus a key regulatory factor in the human type I IFN cascade in myeloid cells and plays an important role in the induction of human innate immune genes (52). In summary, PYHIN family members, particularly IFI16, modulate HBV through DNA recognition, IFN induction, and inflammasome activation (**Figure 3**), but the contributions of individual family members differ and several mechanisms require further experimental validation.

**Figure 3:**
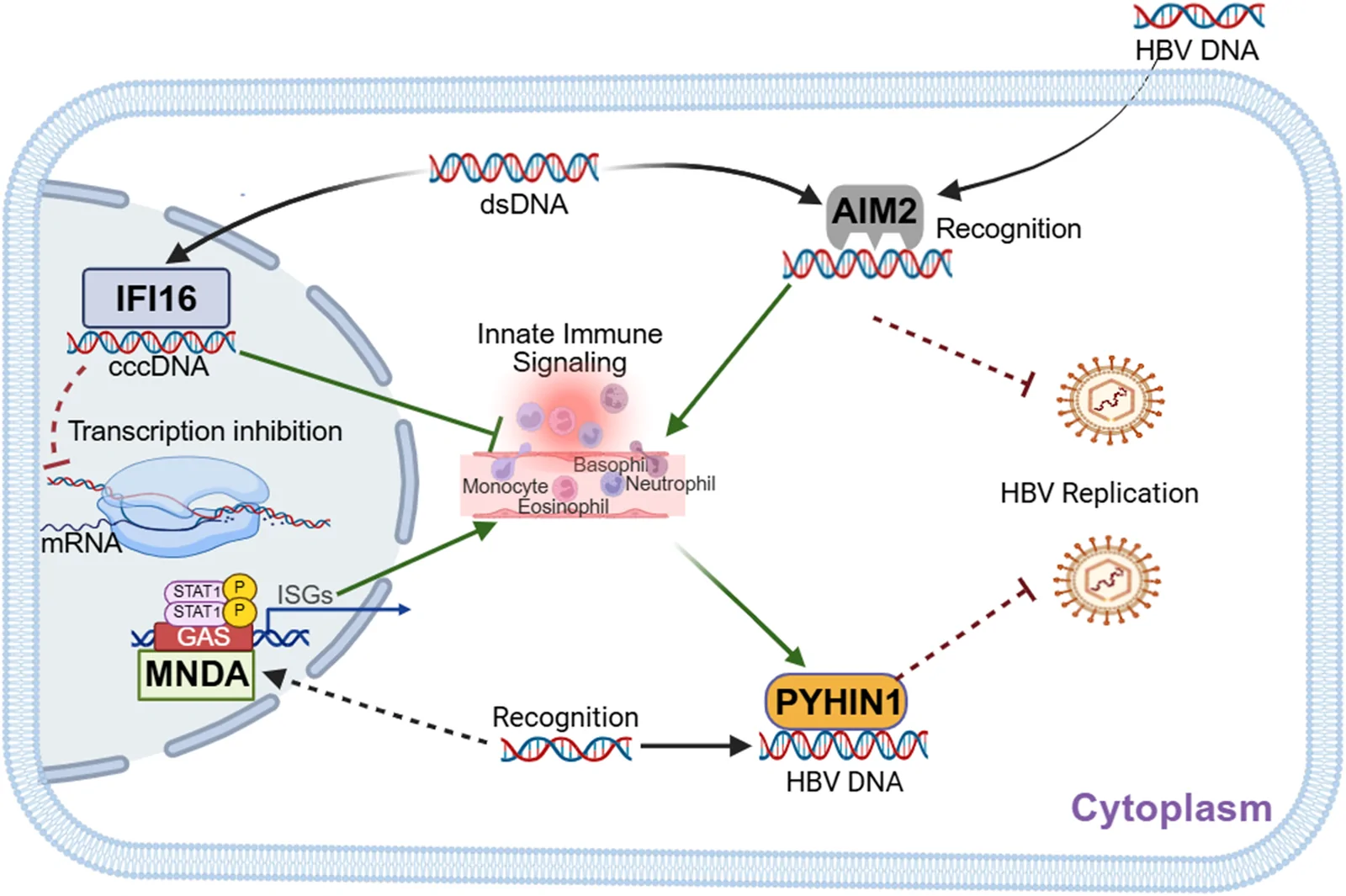
Schematic diagram illustrating the role of host PYHIN family proteins in HBV DNA infection. IFI16 has been proven to be able to recognize and bind to the HBV cccDNA in the cell nucleus, directly inhibiting the transcription of the HBV genome, thereby preventing the generation of viral mRNA; while MNDA does not directly recognize viral DNA to form inflammasomes, it is hypothesized that its function is achieved by regulating the transcription of IRF7. AIM2 and PYHIN1 are considered to trigger the host innate immune signaling pathway by recognizing viral dsDNA in the cytoplasm, ultimately exerting the effect of inhibiting HBV replication. Solid lines represent the pathways verified experimentally in the HBV system. Dashed lines represent the mechanisms inferred based on other virus or indirect evidence. This image was created using BioRender.

### 3.3 Direct inhibition of HBV transcription and replication

IFI16 not only recognizes nuclear DNA, but also directly inhibits HBV transcription of host genes. IFI16 can bind to IFN sensing elements in cccDNA and recruit epigenetic regulation, resulting in decreased acetylation of histone H3, increased H3K27me3, and conversion of cccDNA into chromatin with transcriptional repression, thereby reducing HBV RNA synthesis and viral replication (40, 43, 53). IFI16 can also interact with host factors such as DDX5 to form complexes, thereby enhancing the silencing process of cccDNA, which is consistent with its role in transcriptional regulation (54). PYHIN1 is a broad-spectrum antiviral restriction factor that sequesters Sp1 to suppress HIV-1 viral gene expression, a mechanism shared with IFI16 and MNDA (15). However, these findings derive mainly from overexpression systems *in vitro* (12, 15), and direct antiviral activity of endogenous PYHIN1 against HBV in physiologically relevant hepatocyte models remains to be validated.

## 4 Viral evasion of PYHIN-mediated immunity

### 4.1 HBx-mediated suppression of PYHIN sensors and signaling

HBV inhibits host DNA-sensing function and the corresponding downstream signaling pathways through various strategies, thereby weakening innate immunity mediated by PYHIN. These mechanisms include directly antagonizing the sensing and signaling complexes, immune suppression mediated by viral secreted antigens, as well as the effects of genomic and epigenetic variations (40, 55, 56).

HBV has been shown to directly interact with PYHIN family members, such as IFI16, through its HBx protein, thereby interfering with their antiviral functions (57). HBx is a key regulator by which HBV suppresses host antiviral responses. It impairs DNA sensing pathways and reduces the induction of IFNs and ISGs by promoting degradation, ubiquitination, or autophagic turnover of cGAS, STING, or downstream molecules (56). Accordingly, HBx can indirectly inhibit amplification of the IFI16-STING signaling pathway, although this effect is mediated through shared downstream components rather than direct antagonism of IFI16 itself, direct molecular interactions between IFI16 and HBx require further experimental validation (34, 40, 58, 59). HBx has been shown to downregulate AIM2 expression in hepatoma cell lines (58). While this downregulation has been proposed to contribute to viral immune evasion and liver immunopathology, the extent to which it constitutes a physiologically relevant HBV immune-evasion strategy has not been conclusively demonstrated, as the current evidence derives primarily from *in vitro* hepatoma cell-line models rather than from physiologically relevant HBV infection systems (58, 60). In addition, HBV can promote the degradation of certain PYHIN proteins via the ubiquitin-proteasome or autophagy-lysosome pathway, further dampening host DNA sensing (61). Whether this down-regulation represents an immune evasion strategy during HBV infection still requires further direct verification.

### 4.2 Immunomodulatory roles of HBsAg and HBeAg

The secreted antigen HBsAg interferes with dendritic cell and macrophage function, suppresses RIG-I/MAVS and related PRR pathways, reduces type I IFN production, and alters downstream TBK1/IRF3 activity (10). Collectively, these effects suppress intracellular DNA sensing at the microenvironmental level (59), although this might reflect an indirect regulation of the innate immune environment rather than a direct antagonistic effect on the PYHIN sensor.

HBeAg plays a significant role in establishing immune tolerance. It suppresses Toll-like receptor signaling and reduces proinflammatory cytokine production, thereby indirectly limiting activation of intracellular DNA sensors such as IFI16 and AIM2 (59, 60). Clinical and *in vivo* data further suggest that HBsAg and HBeAg promote viral persistence by modulating the immune microenvironment and inhibiting inflammasome activation (59).

### 4.3 Evasion via cccDNA cloaking and viral mutations

cccDNA exists as a minichromosome bound to host histones. Histone modifications and DNA methylation reduce its transcriptional activity and render its structure more similar to host chromatin, thereby potentially decreasing the likelihood of recognition by nuclear DNA sensors (62, 63). Moreover, rcDNA is rapidly repaired into cccDNA upon nuclear entry, minimizing the duration of exposed viral DNA in the cytoplasm and reducing opportunities for detection by cytosolic DNA sensors such as AIM2 and cGAS (44, 64). HBV genomic variation and CpG site methylation may alter viral DNA conformation or accessibility, thereby affecting the binding affinity and recognition efficiency of DNA-binding proteins such as IFI16. Current evidence indicates that CpG methylation correlates with viral transcriptional activity (62, 65). However, a direct causal relationship between CpG methylation and reduced PYHIN recognition has not been established, so the mechanism needs to be further verified in the future.

Overall, HBV has been proposed to evade the PYHIN family-mediated immune response through four synergistic mechanisms. Firstly, there is a direct protein antagonistic effect: HBx disrupts the cGAS/STING/TBK1/IRF3 axis and downregulates AIM2; secondly, it achieves microenvironmental inhibition by secreting HBsAg/HBeAg antigens, hindering PRR signal transduction and the functions of dendritic cells and macrophages indirect effects on DNA sensing; thirdly, it also has an epigenetic concealment mechanism, including rapidly converting rcDNA to cccDNA to reduce the exposure of cytoplasmic DNA; finally, it may also do so through genomic and epigenetic variations. Although some direct experimental evidence supports some of the mechanisms, it should be noted that the majority of current mechanistic studies rely on transformed cell lines or overexpression systems, which may not fully recapitulate the conditions of natural HBV infection. Validation in physiologically relevant models will be essential. systematic research is still needed to understand the specific roles and relative contributions of each PYHIN member during acute and chronic HBV infections in patients (56, 60, 65).

## 5 Dual pathophysiological roles of PYHIN activation in chronic HBV infection

### 5.1 PYHIN-mediated antiviral defense and chronic liver injury

Although PYHIN proteins contribute to antiviral defense, HBV can attenuate this response through multiple immune-evasion mechanisms. Suppression of PYHIN expression weakens DNA sensor-mediated innate immunity and may create a cellular environment that favors viral persistence (58, 66).

PYHIN-dependent inflammatory signaling may represent a point of divergence in chronic HBV infection, where pathways that contribute to antiviral defense could also sustain tissue-damaging inflammation when activation persists. This dual role has been linked conceptually to hepatic fibrosis and HCC development, but direct evidence from HBV infection models remains sparse, and the framework is primarily inferred from studies of other chronic liver diseases or infections caused by other DNA viruses (12, 67). AIM2 provides a clear example of this evidence gap. In non-HBV models of hepatic injury, persistent AIM2 inflammasome activation induces caspase-1-dependent release of IL-1β and IL-18, thereby intensifying hepatic inflammation and fibrogenesis (20, 48). However, whether AIM2 drives comparable inflammatory or profibrotic programs during HBV infection has not been directly established.

Compared with AIM2, IFI16 is supported by more direct evidence in HBV-associated inflammatory liver disease. Specifically, IFI16 expression is significantly correlated with inflammatory severity in patients with chronic HBV and HBV-related liver failure (53). Liver tissue from affected patients also contains greater numbers of IFI16-positive cells, with cell abundance positively associated with the degree of inflammation, supporting involvement in immune-mediated hepatic injury (20). At the mechanistic level, IFI16 activates the caspase-1/IL-1β axis and may promote apoptosis or pyroptosis, providing plausible routes through which sustained signaling could aggravate tissue damage (60, 68, 69). In a chronically inflamed hepatic microenvironment, persistent IFI16 activation may also induce a senescence-associated secretory phenotype, further reinforcing inflammatory signaling and accelerating disease progression (8, 70). Although AIM2 and IFI16 have both been associated with HCC, current evidence has not established whether PYHIN dysregulation during chronic HBV infection contributes directly to hepatocarcinogenesis or instead reflects broader inflammatory and tumor microenvironment-associated changes.

### 5.2 Genomic instability and tumor-promoting potential

Chronic inflammatory activity in HBV-associated liver disease creates a persistent oxidative and genotoxic environment. Inflammasome activation can amplify cytokine-driven inflammatory circuits and reactive oxygen species (ROS), production, thereby increasing DNA damage and genomic instability, both central contributors to HBV-related hepatocarcinogenesis (71–73). Inflammation-induced oxidative stress further promotes mutation accumulation, chromosomal instability, and oncogenic pathway activation, creating conditions that promote malignant transformation of hepatocytes (71). In addition to canonical roles in innate immune sensing, PYHIN family members also participate in cell-cycle regulation, DNA damage responses, and tumor suppressor pathways (37, 74). IFI16 expression is dysregulated across multiple tumor types, and functional outcomes appear highly context dependent. IFI16 can support tumor-suppressive programs, but under specific pathological conditions it may also reinforce tumor-associated inflammatory microenvironments, primarily through inflammasome activation (75, 76). In HBV-related HCC, persistent inflammation and DNA damage may therefore alter the functional output of PYHIN proteins such as IFI16, shifting them from antiviral effectors toward pro-tumorigenic inflammatory mediators (8, 71).

### 5.3 Clinical evidence and translational implications of PYHIN signaling

Clinical observations support a link between hepatic IFI16 expression and immunopathological activity in chronic HBV infection. In patients with chronic HBV infection, IFI16 expression correlates with inflammatory severity and is significantly associated with disease progression. Altered IFI16 expression has been detected across multiple hepatic cell populations involved in immune regulation, including kupffer cells, endothelial cells, and dendritic cells, indicating potential involvement in shaping the HBV-associated immune microenvironment (20, 47). Notably, IFI16 shows only a relatively weak correlation with HBV DNA load but a stronger association with liver injury markers (20), suggesting that IFI16 may track hepatic injury more closely than viral burden. Clinical evidence for AIM2 remains less developed, although broad activation of inflammasome-related pathways in HBV-associated hepatitis and fibrosis supports potential clinical relevance (20, 50). Studies have linked polymorphisms in innate immune pathway genes to HBV infection outcomes and HCC risk, yet genetic variation within the PYHIN family have not been thoroughly explored in this clinical context (12, 77). Given the emerging roles of PYHIN proteins in antiviral DNA sensing, inflammatory regulation, and tumor biology (11, 26), future work should determine whether PYHIN polymorphisms modify viral clearance, progression to chronic infection, and HCC susceptibility, with potential implications for risk stratification and individualized therapeutic strategies.

Overall, PYHIN proteins appear to exert context-dependent functions in HBV-related liver disease. Through DNA sensing and IFN induction, these proteins can restrict viral replication. With persistent activation, inflammasome signaling and chronic inflammatory amplification may contribute to liver injury, fibrosis, and hepatocarcinogenesis, although direct HBV-specific evidence supporting this pathogenic shift remains limited. The clinical impact of PYHIN signaling is therefore likely determined by the intensity, cellular context, and duration of immune activation (**Figure 4**).

**Figure 4:**
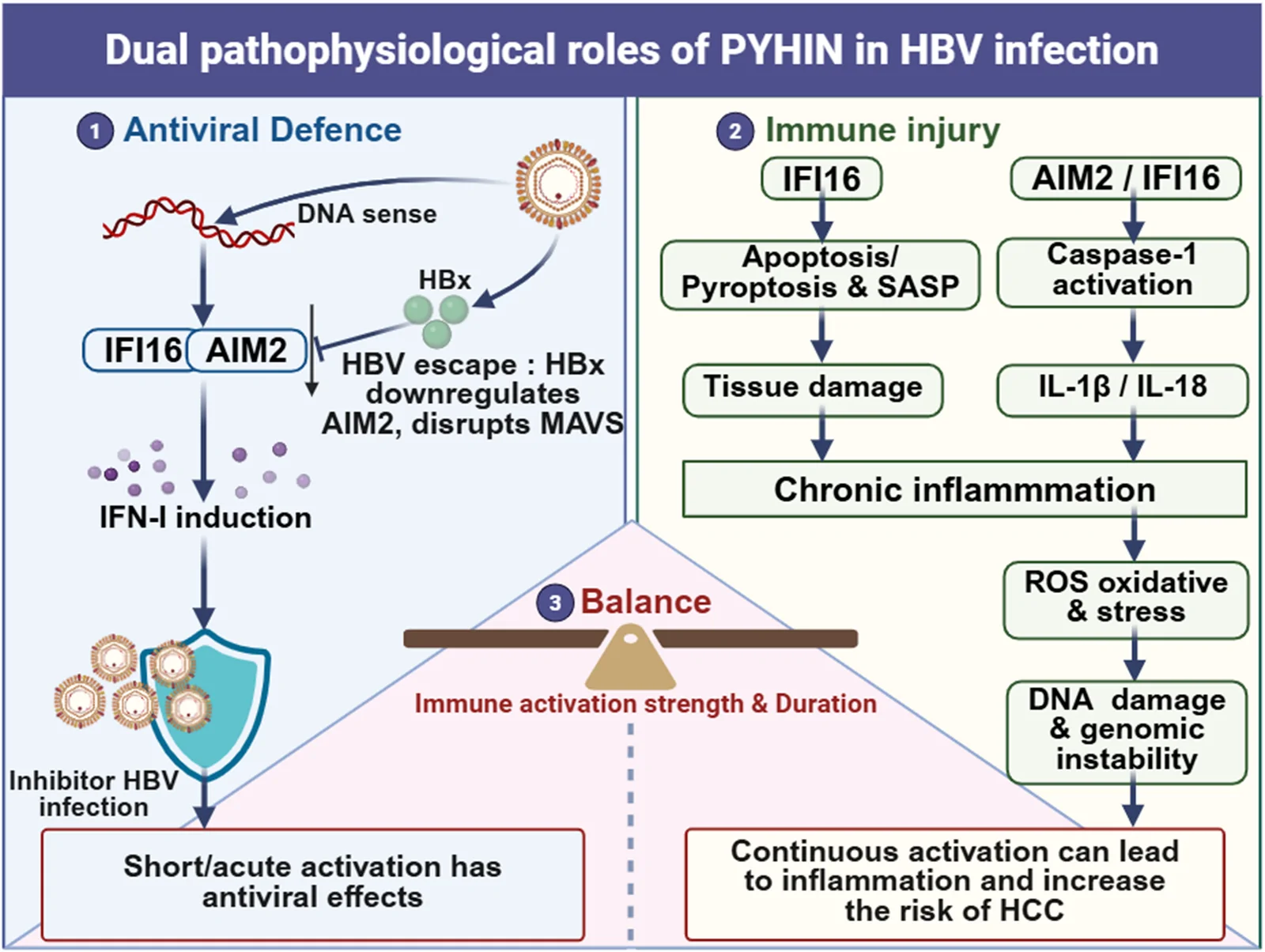
Dual roles of PYHIN protein in HBV infection. IFI16 and AIM2 can detect HBV DNA, thereby triggering interferon-mediated antiviral response. Conversely, the viral HBx protein has been reported to promote immune evasion by downregulating AIM2 expression and disrupting the MAVS-dependent signaling pathway. Upon sustained activation, AIM2 and IFI16 drive the caspase-1 dependent maturation and release of IL-1β and IL-18, which may exacerbate hepatic inflammation and fibrosis. IFI16 has also been associated with induction of SASP, potentially exacerbating tissue damage. The persistent inflammatory signals can produce ROS, leading to DNA oxidative damage and genomic instability, and this mechanism is believed to be related to the occurrence of HCC. In the context of chronic inflammation, it has been proposed that PYHIN proteins may shift from antiviral effector factors to mediators of tumor-promoting inflammation. Thus, the balance between PYHIN antiviral defense and immune injury is thought to depend on the intensity and duration of innate immune activation. MAVS, mitochondrial antiviral signaling protein; ROS, reactive oxygen species; SASP, senescence-associated secretory phenotype. This image was created using BioRender.

## 6 Functional controversies and future research challenges in PYHIN biology

### 6.1 Functional hierarchy and spatial compartmentalization of PYHIN sensors

A central unresolved issue is whether PYHIN sensors function as overlapping antiviral safeguards or occupy distinct positions defined by cell type and subcellular compartment during HBV infection. Recent studies suggest a degree of functional partitioning, with IFI16 acting as a principal nuclear DNA sensor in hepatocytes and AIM2 potentially participating in cytoplasmic DNA sensing in non-epithelial immune cells (12, 59). This proposed division remains difficult to resolve, however, as existing experimental models do not fully capture the cellular and spatial context that governs PYHIN signaling. Emerging data indicate that PYHIN pathway activity is highly plastic and influenced by differentiation state, inflammatory cues, and intracellular localization. IFI16 illustrates this flexibility, as nucleocytoplasmic trafficking may connect transcriptional repression of cccDNA with inflammasome activation (3, 20).

The differences between experimental systems may lead to inconsistent research results. Hepatocyte cell lines usually have defects in innate immune signal transduction. The research results obtained through overexpression systems may exaggerate the function of PYHIN. In contrast, primary hepatocytes and sodium taurocholate cotransporting polypeptide dependent HBV infection models show tighter constraints on PYHIN activation (26, 70). Apparent redundancy in cell-based studies may therefore reflect model-dependent artifacts or unresolved cellular heterogeneity rather than true biological overlap. As such, defining the functional hierarchy of PYHIN sensors will require single-cell and spatially resolved analyses of PYHIN expression, localization, and signaling activity in human liver tissue across distinct stages of HBV infection.

### 6.2 Immune transition of PYHIN signaling from antiviral defense to pathogenic inflammation

Persistent immune activation may shift PYHIN signaling in chronic HBV infection from antiviral surveillance toward inflammatory pathology. IFI16 contributes to antiviral defense through viral DNA sensing and regulation of cccDNA activity (70). In contrast, sustained PYHIN-related inflammasome signaling, particularly through AIM2, has been proposed to maintain IL-1β and IL-18 production, thereby supporting fibrogenic inflammation and HCC-promoting processes. However, direct evidence from HBV infection systems remains insufficient to establish a mechanistic link between PYHIN-mediated inflammasome activation, fibrosis, and HCC (78).

IFI16 may also engage inflammasome-associated cell death programs, including pyroptosis and PANoptosis, with potential amplification of hepatic tissue damage (68, 76). This transition is unlikely to represent a simple temporal sequence from early antiviral defense to late immunopathology. Rather, it may emerge when persistent exposure to HBsAg/HBeAg, ineffective immune clearance, and metabolic or epigenetic reprogramming of hepatic and immune cells uncouple innate immune signaling from viral control. This conceptual framework is consistent with current models of inflammation-driven cancer, in which pathogen-sensing pathways are redirected from host defense toward maintenance of a tumor-promoting inflammatory microenvironment (68, 71). Progress in this area will require models and analytical approaches that preserve or resolve the cellular diversity, immune competence, and spatial architecture of the infected liver, including liver organoids, immune-competent humanized models, and spatial transcriptomics.

### 6.3 Experimental and translational barriers

Despite research progress, mechanistic interpretation of PYHIN function in HBV infection remains constrained by important technical and biological limitations. Selective pharmacological modulators for individual PYHIN proteins are not yet available, limiting, therapeutic development and pathway-level dissection (68). Furthermore, species-specific divergence creates an additional barrier. Mice lack a direct IFI16 ortholog and instead express functionally distinct PYHIN proteins, which restricts the interpretation of murine HBV-related studies for human disease (79). Current models of HBV infection also cannot fully recapitulate the chronic immune-tolerant state or the liver-specific immunopathological processes that shape disease progression, further limiting assessment of PYHIN function in clinically relevant settings (70, 80).

Future studies should prioritize human-relevant systems that preserve the cellular and immunological features of chronic HBV infection. Well-characterized clinical samples, liver-on-a-chip platforms constructed from primary human hepatocytes, and complementary single-cell sequencing approaches will be needed to define how PYHIN signaling is regulated across cell populations, disease stages, and inflammatory states. These approaches should help clarify the specific contribution of PYHIN pathways to viral control, hepatic injury, fibrosis, and disease progression.

## 7 Prospects for PYHIN pathway-targeted therapy

### 7.1 Reinforcing antiviral PYHIN signaling

Controlled activation of PYHIN-mediated antiviral signaling may help restore innate antiviral responses in CHB infection, provided that inflammatory activation is carefully constrained. Activation of IFI16-dependent sensing or the cGAS–STING axis can strengthen intracellular recognition of HBV DNA and promote epigenetic restriction of cccDNA (40, 70). Emerging evidence also indicates that DNA-sensing pathways interact with metabolic regulatory programs and that combined modulation of innate immune signaling and cellular metabolism can improve antiviral efficacy (81, 82). This approach will require precise delivery, because broad activation of DNA-sensing pathways could increase the risk of systemic inflammatory toxicity. Nanotechnology-based platforms that preferentially target hepatocytes or antigen-presenting cells offer a potential strategy for cell-specific activation of DNA-sensing pathways while minimizing systemic toxicity (83, 84). Future PYHIN-oriented therapies will therefore require carefully titrated pathway activation coupled with delivery systems that preserve antiviral efficacy without amplifying inflammatory damage.

### 7.2 Inhibition of PYHIN-driven immunopathological responses

Persistent inflammasome activation has been proposed to contribute to HBV-associated liver immunopathology. AIM2 and downstream inflammatory mediators therefore warrant investigation as therapeutic targets for limiting inflammatory injury and fibrotic progression. Although selective AIM2 inhibitors remain under development, AIM2 and IFI16 have been linked to activation of the caspase-1/IL-1β/IL-18 axis in HBV-relevant experimental settings (60, 68). These findings provide a preliminary rationale for evaluating caspase-1 inhibition or blockade of IL-1β or IL-18 signaling in inflammatory and fibrotic liver disease, although HBV-specific clinical validation is still required (78, 85).

Therapeutic selectivity, however, remains a major challenge. Broad inhibition of inflammasome signaling may weaken host defense and impair antiviral immune control. Effective intervention will therefore require disease-stage- and context-specific modulation that dampens pathological inflammation without abolishing protective antiviral responses. Future work should define the specific AIM2 domains that mediate inflammatory activation, which may support the development of more precise modulators or regulatory strategies.

### 7.3 Progress toward combination and precision therapy

Given the persistence and immunological complexity of HBV infection, interventions targeting PYHIN are unlikely to be effective as stand-alone therapies. A more plausible strategy is to integrate PYHIN modulation with established antiviral and immunomodulatory regimens. Nucleos(t)ide analogs may provide a foundation for PYHIN-directed combination therapy by suppressing HBV replication before immune modulation is introduced. In this setting, controlled activation of PYHIN-related DNA-sensing pathways could be paired with IFNs or STING agonists to strengthen innate antiviral signaling, while coordinated use with immune checkpoint blockade may help restore exhausted antiviral T-cell responses. Epigenetic therapies also warrant investigation, as restoration of PYHIN expression may enhance cccDNA recognition and transcriptional restraint (70). Future studies should integrate host genomic profiling with immune phenotyping to define which patients are most likely to benefit from PYHIN pathway modulation and to guide combination strategies that enhance antiviral control without intensifying inflammatory liver injury.

## 8 Conclusion

As the research progresses, the role of the PYHIN family proteins in the HBV infection process is being continuously elucidated. The translational studies of these proteins may provide new key regulatory drugs for antiviral immunity, inflammation, and tumor treatment. The functions of PYHIN are not limited to the traditional DNA sensing, but also include epigenetic regulation, activation of inflammasomes, and coordination of cell death pathways. Critically, whether the function of the PYHIN protein is protective or pathogenic appears to depend on the context. The results are influenced by the activation intensity, duration and local cellular environment (**Figure 5**). Further mechanism studies are needed to clarify the role of PYHIN members in HBV infection and the underlying regulatory mechanisms, which is crucial for developing new therapeutic strategies based on the PYHIN protein.

**Figure 5:**
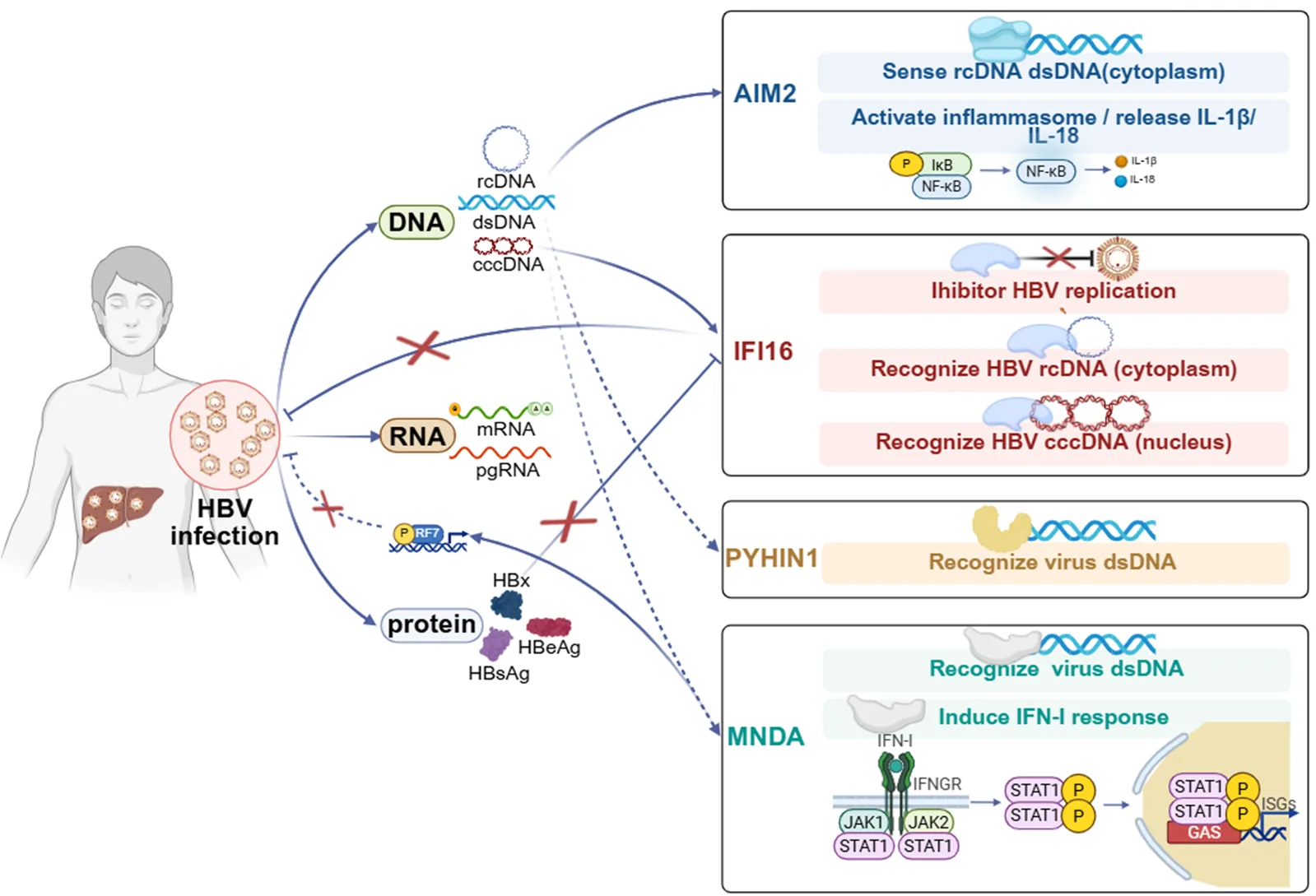
Proposed model of PYHIN protein family-mediated recognition of HBV components and innate immune activation after HBV infection. During the HBV infection process, its nucleic acid and protein components can be recognized by cytoplasmic and nuclear DNA sensors, thereby triggering antiviral immune responses. The replication intermediates of HBV include rcDNA, dsDNA, and cccDNA, as well as pre-genomic RNA, mRNA, and various viral proteins. The host PYHIN proteins AIM2 and IFI16 have been proven to be involved in the recognition of these viral components. Specifically, AIM2 is located in the cytoplasm and activates the inflammasome by recognizing rcDNA and dsDNA, promoting the release of IL-1β and IL-18. IFI16 has a dual spatial localization and can recognize HBV’s rcDNA in the cytoplasm and cccDNA in the cell nucleus, thereby helping to inhibit HBV replication. PYHIN1 is believed to be involved in the recognition of viral dsDNA. MNDA, although not directly recognizing viral dsDNA, is speculated to initiate the type I IFN response by regulating the transcriptional activity of IRF7. Studies have shown that the HBV protein HBx may, through some mechanism, block the recognition and signal transduction of these DNA sensors, thereby possibly helping the virus evade the host’s innate immune defense. Solid lines represent experimentally verified interactions. Dashed lines represent the mechanisms inferred based on other virus or indirect evidence. This image was created using BioRender.
